# Inactivation of group II intron RmInt1 in the *Sinorhizobium meliloti* genome

**DOI:** 10.1038/srep12036

**Published:** 2015-07-09

**Authors:** María Dolores Molina-Sánchez, Nicolás Toro

**Affiliations:** 1 Grupo de Ecología Genética, Estación Experimental del Zaidín, Consejo Superior de Investigaciones Científicas, Calle Profesor Albareda 1, 18008 Granada, Spain

## Abstract

Group II introns are self-splicing catalytic RNAs that probably originated in bacteria and act as mobile retroelements. The dispersal and dynamics of group II intron spread within a bacterial genome are thought to follow a selection-driven extinction model. Likewise, various studies on the evolution of group II introns have suggested that they are evolving toward an inactive form by fragmentation, with the loss of the intron 3′-terminus, but with some intron fragments remaining and continuing to evolve in the genome. RmInt1 is a mobile group II intron that is widespread in natural populations of *Sinorhizobium meliloti,* but some strains of this species have no RmInt1 introns. We studied the splicing ability and mobility of the three full-length RmInt1 copies harbored by *S. meliloti* 1021, and obtained evidence suggesting that specific mutations may lead to the impairment of intron splicing and retrohoming. Our data suggest that the RmInt1 copies in this strain are undergoing a process of inactivation.

*Sinorhizobium meliloti* is a nitrogen-fixing bacterium that establishes a symbiotic interaction with legumes of the genera *Medicago*, *Melilotus* and *Truncatula*. The *S. meliloti* genome carries many different mobile genetic elements, including group II introns, which are self-splicing catalytic RNA and mobile retroelements^1^,^2^. RmInt1 is a mobile group II intron that is widespread in natural populations of *S. meliloti*^3^ and was first described in the GR4 strain^4^. The complete genome sequence of this strain has recently been reported^5^. It contains 10 copies of RmInt1 distributed between the different replicons harbored by this strain, including the chromosome. The number of RmInt1 copies has been shown to differ between *S. meliloti* strains for which complete genome sequences are available, but it has been estimated that 90% of *S. meliloti* isolates harbor this intron and that strains lacking RmInt1, such as RMO17, remain suitable for intron colonization^6^,^7^.

Group II introns consist of a structured RNA that folds into a conserved three-dimensional structure organized into six double-helical domains, DI to DVI^8^. Bacterial group II introns generally have an open reading frame (ORF) encoding an intron-encoded protein (IEP) in DIV that acts as a reverse transcriptase, followed by an RNA-binding domain with RNA splicing or maturase activity, and, in some intron lineages, a C-terminal DNA-binding and endonuclease domain^9^,^10^,^11^. The mobility of group II introns, a process known as retrohoming^1^,^2^,^12^, is based on the formation of a ribonucleoprotein (RNP) complex consisting of the intron-encoded protein (IEP) and the spliced intron lariat RNA, which remains associated with the IEP. The RNP recognizes its intron target via both the IEP and the intron lariat RNA. The exon binding sites (EBS) of the lariat RNA base pair with the intron insertion site of the complementary region of the DNA target, the intron-binding site (IBS), and the target flanking regions are recognized by the IEP. These retroelements are highly specific, recognizing a target of ∼25–30 nts in length, but they are nevertheless able to invade locations other than the usual homing site (ectopic transposition), albeit at low frequency.

The natural target site of RmInt1 lies within the IS630 family insertion sequence IS*Rm2011-2* (IS*Rm11*)^13^, and in other closely related IS elements^14^,^15^,^16^. RmInt1 can also invade the *oxi1* gene^3^,^14^, which carries IBS sequences resembling those present in IS*Rm2011-2*. Unlike RmInt1, this IS has been found in all the *S. meliloti* strains and isolates tested.

It has been suggested that, like other mobile elements, group II introns will follow a colonization/extinction model, with the elimination of highly colonized genomes from the population by purifying selection^17^. However, group II introns may also follow a model of inactivation within the bacterial genome, decreasing the selective pressure on the population. Various studies of the evolution of group II introns in the bacterial genome have suggested a process of evolution toward an inactive form by fragmentation, with the loss of the intron 3′-terminus^18^,^19^. We have also reported that the gradual elimination of group II introns by the host during evolution would be unlikely to result in the complete eradication of intron sequences in all cases, with some intron fragments instead remaining and continuing to evolve within the genome^20^.

We report here the splicing and retrohoming abilities of the three full-length RmInt1 copies of *S. meliloti* 1021, which display minor sequence divergences, but carry common and specific point mutations affecting both the intron ribozyme RNA and the IEP. We identified unanticipated specific mutations leading to the inactivation of intron splicing and retrohoming. Our results suggest that the RmInt1 copies of strain 1021 are undergoing a process of intron inactivation.

## Results

### The splicing ability of the RmInt1 copies of *S. meliloti* 1021

**Figure 1:**
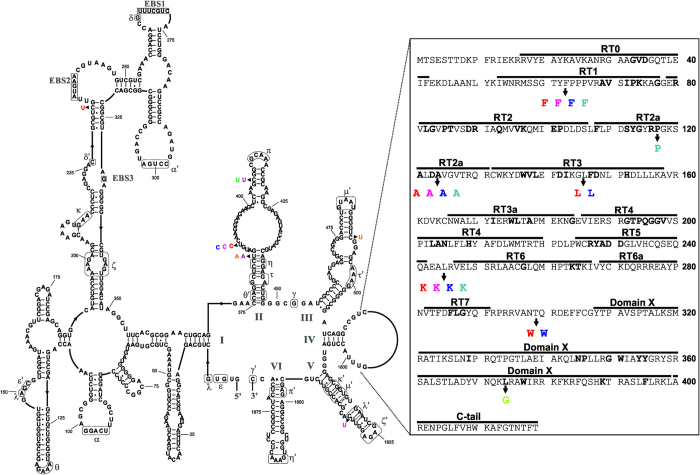
RmInt1 copies in *S.* *meliloti*. The predicted secondary structure of the RmInt1 ribozyme and the amino-acid sequence of the intron-encoded protein (IEP) are shown. Roman numerals indicate the six stem-loop domains described for group II introns (DI to DVI), and boxed residues identify exon binding sites 1, 2 and 3 (EBSs). Long-range interactions are indicated by dashed, gray lines and Greek letters. The boxed sequence emerging from DIV corresponds to the IEP. Residues highly conserved among group II introns are shown in bold, with the different domains of the IEP marked above the sequence. RmInt1 point mutations identified in different strains of *S. meliloti* are highlighted with a color code: S.me_1021_pA_1 and S.me_2011_pA_1, in red; S.me_1021_pB_1 and S.me_2011_pB_1, in fuchsia, S.me_1021_pB_2 and S.me_2011_pB_2, in blue; S.me_GR4_pC_1 and S.me_GR4_pC_4, in purple; S.me_GR4_pC_3, in green; the six copies in *S. meliloti* SM11 are identified in light blue; the four copies in *S. meliloti* BL225C, in orange; and finally, S.me_102F51_pB_1 copy is shown in light green.

As previously reported, the *S. meliloti* 1021 genome contains five full-length (2 in pSymA and 3 in pSymB) and one fragmented group II intron (in pSymB)^15^. Three of these full-length introns are copies of RmInt1 (hereafter referred as to *S. meliloti* GR4 RmInt1 sequence deposited in GenBank accession n° Y11597), diverging only slightly in sequence (99% identical): SMa1801, SMb21708 and SMb21699 (hereafter referred as to S.me_1021_pA_1, S.me_1021_pB_1, and S.me_1021_pB_2, respectively; see table 1). The sequence differences between these introns concern several domains in the ribozyme (DI, DII and DV) and mostly correspond to silent mutations in the intron-encoded protein sequence (DIV, Fig. 1). The three RmInt1 variants of strain 1021 display a U-to C-transition at position 389, which is located in the major loop region of DII. They also carry two silent mutations (F63 and A124) and a conservative missense mutation (R246K) in the ORF. S.me_1021_pA_1 and S.me_1021_pB_2 have two additional coding sequence mutations: a silent mutation affecting domain 3 of the reverse transcriptase (L146) and another preceding the maturase domain (R301W). Furthermore, S.me_1021_pA_1 displays a C-to-U nucleotide exchange at position 232, with no apparent effect on secondary structure because base-pairing to the G residue in position 323 is conserved. Similarly, a C1832-G1819 base pair has been replaced by a U◦G pair, just before the unpaired region of DV in the S.me_1021_pB_1 ribozyme.

**Figure 2:**
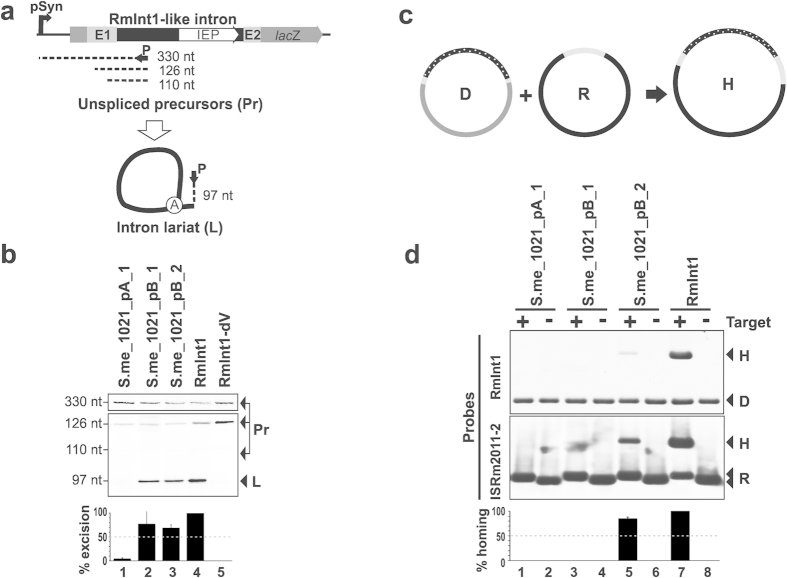
Splicing and retrohoming of RmInt1-like introns in *S.* *meliloti*. RMO17 (**A**) The primer extension products is shown. The black box represents the intron ribozyme, and a white arrow indicates the intron-encoded protein. Flanking gray boxes correspond to the exon sequences, and the Syn promoter is also indicated. The circled A identifies the bulged adenosine in DVI. A black arrow indicates oligonucleotide P, which was used for primer extension and the dashed lines identify the resulting cDNA products. Sizes are indicated in nucleotides. cDNA products of 110 and 126 nt are derived from unspliced precursor transcript processed at specific 5´U residues^33^ and the 330 nt corresponds to the entire transcript. (**B**) Total RNA from *S. meliloti* RMO17 harboring plasmids expressing the RmInt1-like introns described in *S. meliloti* 1021 (S.me_1021_pA_1, S.me_1021_pB_1 and S.me_1021_pB_2) was reverse-transcribed with an intron-complementary primer (P). RmInt1 was used as the positive control, and a splicing-defective ribozyme domain V (DV) mutant was used as the negative control. The 97-nt cDNA (L) products derived from excised intron RNA, and more slowly migrating bands corresponding to unspliced precursors (Pr, 330, 126 and 110 nt) are indicated. Excision rates are plotted at the bottom, as a percentage of RmInt1 splicing levels. (**C**) Mobility assay (not drawn to scale). D represents the donor plasmids; R, the recipient plasmids; and H, the homing plasmids. Gray lines identify the exon sequences and spotted lines correspond to the intron sequences. (**D**) The plasmid pools from *S. meliloti* RMO17 carrying the recipient (pBB0.6) and various donor (pICG derivatives) plasmids were analyzed by *Xba*I digestion and Southern hybridization. A recipient plasmid lacking the DNA target site (pBBΔ129) was used as a negative control. Transposition events were detected with probes specific for the intron ribozyme (RmInt1, upper panel) or the target DNA (IS*Rm2011-2*, lower panel). The hybridization bands corresponding to the donor plasmid (D), the target proficient (+) or deficient (-) recipient plasmids (R) and the homing product (H) are indicated. Retrohoming levels were evaluated as a percentage of the RmInt1 homing rate and the resulting graph is shown below the hybridization panels.

We previously showed that RmInt1 expressed from a plasmid was splicing-competent in the genetic background of *S. meliloti* 1021^15^. However, intron excision rates were significantly lower than the splicing rate observed in *S. meliloti* RMO17. We investigated whether any of the three copies of RmInt1 from *S. meliloti* 1021 were active introns, by analyzing their ability to excise in the *S. meliloti* RmInt1-less strain RMO17. We performed primer extension analysis on RNA isolated from RMO17 harboring the corresponding pICG intron-donor plasmids. Reverse transcription was achieved with a primer complementary to positions 80-97 in RmInt1 DI (Fig. 2A). RmInt1, and its splicing-deficient derivative RmInt1-DV, which contains a mutation of the catalytic triad in DV, were used as the references for splicing rate (Fig. 2B, lanes 4 and 5). The introns originally located in pSymB (S.me_1021_pB_1 and S.me_1021_pB_2) had slightly lower excision rates than RmInt1 (Fig. 2B, lanes 2 and 3). However, the third copy found in *S. meliloti* 1021 pSymA (S.me_1021_pA_1) displayed almost no splicing (Fig. 2B, lane 1). These results are consistent with a core contribution of the C232-G323 base pair preceding the EBS2 loop to RmInt1 maturation, all the other point mutations being tolerated and having only minor effects on intron excision.

### The mobility of the RmInt1 copies of *S. meliloti* 1021 is impaired

By contrast to our findings for splicing, there was strong evidence to suggest that intron mobility was impaired in *S. meliloti* 1021, with mobility rates much lower than those for strain GR4, which has 10 RmInt1 copies distributed between its five replicons, or the intron-less strain RMO17^15^,^21^. It has been suggested that the final steps in RmInt1 retrohoming are impaired in *S. meliloti* 1021, potentially affecting functions essential for the completion of the process^21^. We increased the sensitivity of analyses of invasion rates for *S. meliloti* 1021 RmInt1 variants, by using a two-plasmid system in *S. meliloti* RMO17 (Fig. 2C). The donor constructs express the intron under the control of the constitutive promoter Syn^22^, whereas the target site was present in pBB0.6^23^. The reference value for homing rate was provided by RmInt1 (Fig. 2D, lane 7). A homing product (H) was detected only in the plasmid samples extracted from *S. meliloti* RMO17 harboring pICG_S.me_1021_pB_2 (Fig. 2D, lane 5). No homing products were observed for the remaining introns (lanes 1 and 3). Moreover, the retrohoming efficiency of S.me_1021_pB_2 was slightly lower than that of RmInt1, possibly due to the lower splicing activity of this intron copy. Remarkably, the mobility of S.me_1021_pB_1, which was excised at a similar rate to S.me_1021_pB_2, was completely inhibited. The point mutation at position C1832 therefore seems to abolish the reverse splicing ability of the intron, but not its forward splicing.

### Point mutations inactivate RmInt1 in *S. meliloti* 1021

**Figure 3:**
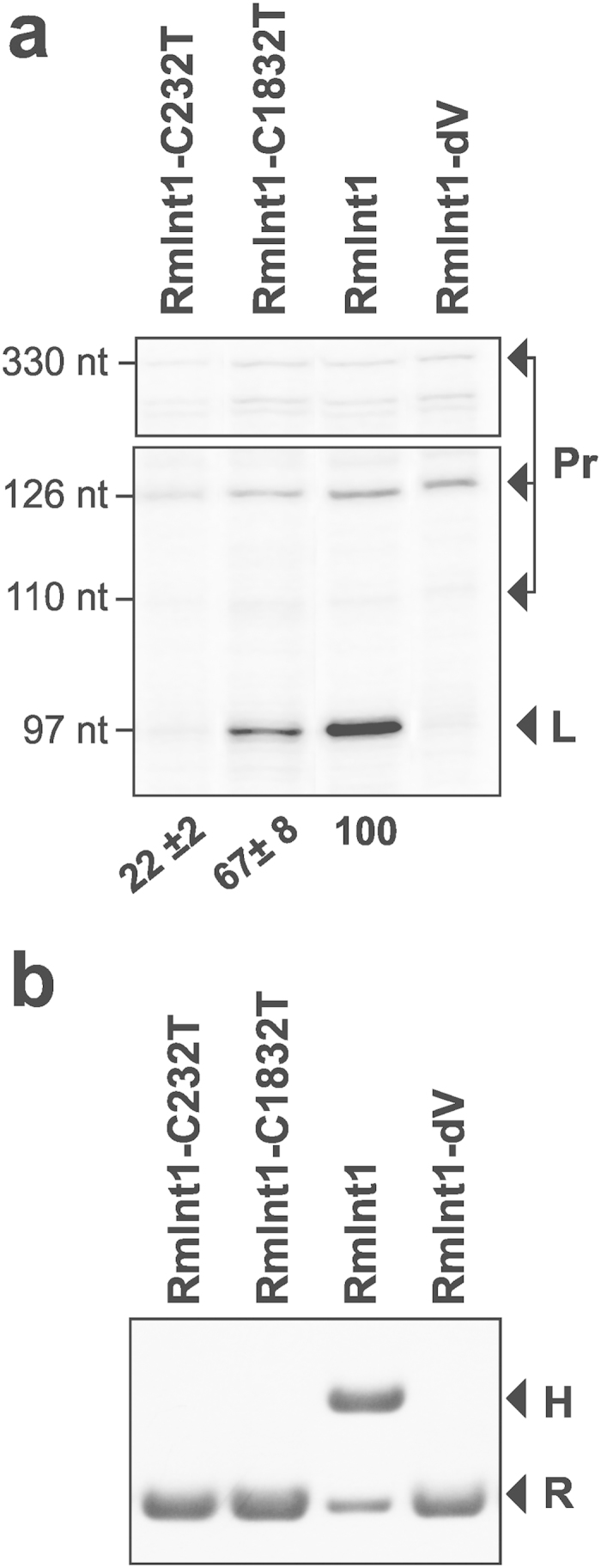
Splicing and retrohoming *in vivo* of RmInt1 mutant introns in *S.meliloti*. RMO17 (**A**) Total RNA from *S. meliloti* RMO17 carrying plasmids expressing RmInt1 mutant constructs (C232U or C1832U) was reverse-transcribed with an intron-complementary primer (P). The cDNA corresponding to the excised intron (L, 97-nt) and unspliced precursors (Pr, 330, 126 and 110 nt) are indicated. RmInt1 was used as the positive control, and the splicing-defective DV mutant was used as a negative control. The excision rates are indicated below the gel and are expressed as a percentage of the splicing efficiency of RmInt1 (taken as 100%). (**B**) The plasmid pools from *S. meliloti* RMO17 harboring the recipient (pBB0.6) and the various donor (pICG derivatives) plasmids were analyzed by *Xba*I digestion and Southern hybridization. Retrohoming events were detected with a probe specific for the target DNA (IS*Rm2011-2*). The hybridization bands corresponding to the recipient plasmid (R) and the homing product (H) are indicated.

As the RmInt1 intron copies in strain 1021 have accumulated additional mutations, we introduced point mutations at positions C232 and C1832 into the reference RmInt1 from strain GR4, to determine whether these changes were responsible for the impairments of splicing and intron retrohoming, respectively, in this strain (Fig. 3). The replacement of C232 with a U residue greatly decreased splicing efficiency, as assessed by primer extension (Fig. 3A, lane 1). This defect may be directly related to the abolition of retrohoming in this mutant (Fig. 3B, lane 1). By contrast, the C1832 to U transition mutation decreased the rate of RmInt1 splicing by 33% (Fig. 3A, lane 2). We were unable to detect any homing events for this mutant (Fig. 3B, lane 2). Thus, point mutations at both positions gave results consistent with previous findings, indicating that the observed effects were due to the specific intron mutations studied. These results suggest that the RmInt1 copies in *S. meliloti* 1021 are probably undergoing an inactivation process.

### RmInt1 sequence variation between *S. meliloti* strains

We investigated whether the full-length RmInt1 sequences harbored by the various strains of *S. meliloti* had any mutations in common (Table 1), by aligning nucleotide sequences with identities of 99 to 100% (Supplementary Fig. 1).

The three intron copies in *S. meliloti* strains 1021 and 2011 were identical, displaying the nucleotide changes described above (Fig. 1). Seven of the 10 RmInt1 copies in *S. meliloti* GR4 were identical, including that used as the reference sequence (Y11597). Thus, only three introns presented two types of variation (all affecting the ribozyme DII) with respect to the reference RmInt1 sequence: all three (S.me_GR4_pC_3, S.me_GR4_pC_1 and S.me_GR4_pC_4) had a T residue in the C407 position, corresponding to the main loop of DII, and S.me_GR4_pC_1 and S.me_GR4_pC_4 had an additional mutation converting the T384 residue into an A residue, thereby disrupting the Watson-Crick U-A pairing at the upper site of the proximal stem of DII. The five RmInt1 copies annotated in *S. meliloti* BL225C were identical and displayed a single T-to-A transversion in position 384. Similarly, the T-to-C transition at position 735, in DIV, was common to three copies in strain 1021 and the five copies present in SM11, which also displayed four additional changes: residue C492T (DIII), G897A, T918C and G1283A, these last three changes affecting DIV. The T918C and G1283A point mutations were also found in the three RmInt1 copies of strain 1021. The only copy found in *S. meliloti* Rm41 was identical to RmInt1, whereas the copy located in pSymB in *S. meliloti* 102F51 presented two nucleotide exchanges (C638G, and C1673G in DIV). Our results indicate that there are strain-specific, and common mutations in the RmInt1 copies harbored by different *S. meliloti* strains, and that some of these mutations may lead to intron inactivation.

## Discussion

We studied the splicing and retrohoming capabilities of the three full-length RmInt1 intron copies harbored by *S. meliloti* strain 1021, and showed that this strain is undergoing a process of inactivation. We identified specific nucleotide exchanges that have limited intron functionality. The replacement of the C residue in position 1832 in DV with a U residue had only a slight effect on intron excision, but abolished mobility. By contrast, replacement of the C residue in position 232 in DI with a U residue resulted in the severe impairment of both forward and reverse splicing. A comparison of the sequences of the full-length RmInt1 copies present in *S. meliloti* strains revealed that some carried strain-specific and common mutations potentially driving intron inactivation.

Group II introns are currently seen as selfish genetic elements that have survived in bacteria due to their preferential retrotransposition to sites outside of functional genes, generally within intergenic regions or other mobile genetic elements. Group II introns are thought to follow a colonization/extinction cycle similar to that described for other mobile genetic elements^17^. This hypothesis appears to be applicable to the introns of *S. meliloti*, in which the RmInt1 intron has spread very successfully, due to the high abundance of its usual target site, the IS*Rm2011-2* insertion sequence. However, RmInt1 is absent from 10% of *S. meliloti* strains and isolates, and *S. meliloti* RMO17 is the best characterized intron-less strain^6^,^7^. Despite lacking RmInt1, RMO17 harbors other types of intron (Toro, unpublished) and displays no restriction to the functionality and colonization of its genome by acquired mobile RmInt1^6^. By contrast, other *S. meliloti* strains, such as GR4 harbor many RmInt1 copies, at least some of which are highly mobile^4^. Interestingly, we also found other strains with smaller numbers of intron copies, such as *S. meliloti* 1021, in which the spread of the intron throughout the genome appears to have been restricted by point mutations affecting mobility and excision activities.

The catalysis of group II introns clearly requires functional ribozyme domains I and V^24^. Domain V, which contains the catalytic triad AGC and the Mg^2+^ coordination bulged nucleotides, is the most conserved element of group II introns^25^. The unpaired region of domain V in RmInt1 is flanked by a U◦G pair at the proximal stem and a G-C base pair at the distal stem of the hairpin, resulting in a secondary structure resembling that generally suggested for group II introns^26^. This G-C base pair and the paired residues immediately preceding it are part of the λ-λ’ long-range interaction (Fig. 1), which links the upper stem of domain V to a looped region in domain I and the 5′ splice site (G5) in the ai5γ intron^27^. Recent crystallographic studies of the *Oceanobacillus iheyensis* group IIC intron revealed that the nucleotide preceding the bulged residues of domain V forms part of the z-anchor structure responsible for maintaining catalytic core integrity and is also involved in metal coordination^28^,^29^,^30^. Consequently, any change to residue C1832 could result in distortions to the structure of domain V that might block the activities of RmInt1. Intriguingly, reverse splicing is completely abolished, but the excision capacity of the intron is conserved. We conclude from this that this residue must be critical for positioning the 5′ end of the intron in the catalytic core, to attack either the 3′ splice site for excision or the exon junction in the target during retrohoming. It therefore appears likely that we observed an impairment of the second step of splicing that could not be distinguished on primer extension experiments, but that is directly related to homing inhibition, given the decrease in the efficiency of the first step of reverse splicing.

The defect in splicing and intron mobility observed in the C232U mutant was particularly striking. A G-C base pair is conserved at the same site in the organellar intron ai5γ, despite the presence of different flanking residues in the stem^27^. By contrast, the ID(iv) stem is identical to that described for the *P.li.LSUI2* intron^31^. However, no particular function has ever been attributed to the penultimate base pair preceding the EBS2 loop. The weaker interactions around this region suggest possible destabilization of the local structure, potentially leading to misrecognition of the EBS2 sequence. A previous study showed that the EBS2-IBS2 interaction was not absolutely required for RmInt1 intron excision, whereas intron retrohoming involved not only this pairing, but also the EBS1-IBS1 and EBS3-IBS3 interactions^32^,^33^. There is, therefore, no obvious explanation for the strong decrease in excision efficiency observed in this mutant. Further investigations are required to elucidate the key role of this residue in intron activities.

Strains GR4, 1021 and RMO17 may be at different stages of the intron colonization/inactivation/extinction cycle. RMO17 appears to be at “time zero” ready for colonization, whereas GR4 seems to be close to the end of the process of colonization of available target sites by introns and the start of intron inactivation, and 1021 seems to be at the inactivation step.

## Methods

### Bacterial strains and growth conditions

The splicing and homing assays were carried out in *S. meliloti* RMO17 derivatives harboring the corresponding plasmids, and genomic DNA from *S. meliloti* 1021 was used for the amplification of intron copies. *Escherichia coli* DH5α was used for plasmid construction. *S. meliloti* strains were cultured at 28 °C on TY medium^34^, whereas *E. coli* was grown on LB medium at 37 °C. When required, growth media were supplemented with selective concentrations of different antibiotics: tetracycline at a concentration of 10 μg·ml^−1^; and kanamycin at 200 μg.ml^−1^ for rhizobia and 50 μg.ml^−1^ for *E. coli*.

### Plasmid constructs

pICG plasmids are derivatives of pMP220, to which the constitutive Syn promoter has been added upstream from the *lac*Z gene and have been described elsewhere as intron donor constructs^22^. We cloned the RmInt1-like intron sequences found in *S. meliloti* 1021 by successive PCRs with total DNA from *S. meliloti* 1021 as a template. As there is only one copy of ISRm10-1 in the whole *S. meliloti* 1021 genome, S.me_1021_pB_1 was amplified directly with the LMS7 (5′-GGGAGCTCCTAGGGCCGGGGTGAGTAGGCCG-3′) and LMS8 (5′-GGGAGCTCACGTGGCTGATCTTCATCGATGAAA-3′) primers, which include *Bln*I and *Bbr*PI restriction endonuclease sites, respectively. Likewise, the two introns inserted into IS*Rm2011-2* were amplified from the flanking regions with the oligonucleotide primers LMS1 (5′-ATCACACTGCTGCATCTCAGTCGTC-3′) and LMS2 (5′-CTGCAGCTCTTGTGAGTACGCAGGC-3′) for S.me_1021_pA_1, and LMS5 (5′-GAGCCCGTGATTTGCCTCTCATCC-3′) and LMS6 (5′-CTCTTCCTTAGATCGGGGCAGGAGC-3′) for S.me_1021_pB_2. The flanking exons were shortened further by PCR with the primers BlnSac (5′-GGGAGCTCCTAGGCCAGGGGTGAGTAGGCCGG-3′) and SacBbr (5′-GGGAGCTCACGTGCCTCGTTTTCATCGATGAGA-3′). The final products were inserted into pICG2 as *Avr*II/*Pml*I fragments and their integrity was confirmed by sequencing. For homing assays, the pBB0.6 recipient plasmid is a derivative of pBBR322 carrying a DNA fragment derived from IS*Rm2011-2* spanning the intron homing site from −175 to the +466 position and its target-deleted (deletion spanning from −125 to the +4 position of the homing site) derivative pBBΔ129 was used as a negative control^23^.

### Isolation of *S. meliloti* genomic DNA

DNA was extracted with the RealPure genomic DNA extraction kit (Durviz, S.L.U., Valencia, Spain), according to a modified version of the manufacturer’s instructions. Briefly, 6 ml of a saturated bacterial culture was centrifuged at 10,000 x g and the pellet was washed with 0.1% L-laurylsarcosine in TE buffer pH 8. Cells were lysed by incubation for 5 minutes at 80 °C, and the resulting lysate was treated with “RNAse solution” at 37 °C for 60 minutes. The proteins were removed by adding a high-concentration salt solution and the DNA was precipitated with cold 2-propanol. The dried DNA pellet was resuspended in a final volume of 100 μl of MilliQ water. Purified DNA was quantified spectrophotometrically (Nanodrop, PeqLab, Erlangen, Germany). Highly pure genomic DNA (*A*_260_/*A*_280_ ratio of 1.8–2.0) was obtained at a final concentration of about 1.5 μg/μl.

### Total RNA extraction from *S. meliloti*

Total RNA was isolated from cultures of free-living *S. meliloti* RMO17 containing plasmids that express reference or mutant RmInt1, as previously described^35^. Briefly, exponentially growing bacterial culture (6 OD_600_ units) was harvested and washed with 0.1% L-laurylsarcosine in TE buffer pH 8. Bacterial lysis were carried out by adding 600 μl of lysis buffer (1.4% SDS, 4 mM EDTA pH8, 0.4 mg/ml proteinase K) and samples were incubated at 65 °C for 10 minutes. 300 μl of NaCl 5 M were then added to the lysed cells and incubated 10 minutes on ice. The lysates were cleared by centrifugation at 10,000 xg and 4 °C. The supernatants were treated with cold ethanol 100% to precipitate the nucleic acids. After sedimentation by centrifugation, samples were digested with 50 units of DNAse I (Roche) to remove all the DNA traces. Samples were further cleaned by phenol extraction and precipitated with cold ethanol. Finally, RNA preparations were resuspended in 20 μl of RNAse-free water. RNA concentration was determined on a Nanodrop spectrophotometer, RNA quality was checked by gel electrophoresis and RNA purity was evaluated by PCR.

### Primer extension

Primer extension reactions were carried out with oligonucleotide P, as previously described^36^. Samples were resolved by electrophoresis in a denaturing 6% polyacrylamide gel. cDNA bands corresponding to the resolved extension products, spliced intron RNA (L) and unspliced precursors (Pr) were quantified with the Quantity One software package (Bio-RAD Laboratories) and intron splicing was measured as 100 [L/(L+Pr)]. Splicing efficiency was plotted as percentage of wild-type values (reference RmInt1).

### Homing assays

RmInt1 mobility in *S. meliloti* RMO17 was assessed by Southern hybridization, with a two-plasmid system^23^. Plasmids were mobilized from *E. coli* DH5α into *S. meliloti* by tri-parental mating^37^. We used pICG27^22^ and derivatives containing the full-length intron flanked by −20/+5 exons as donor plasmids. The recipient plasmid was pBB0.6, a pBBR1MCS2 derivative carrying a 640 bp fragment of IS*Rm2011-2* including the intron insertion site^23^. As a negative control, *S. meliloti* RMO17 was cotransformed with pICG derivatives and pBBΔ129, which lacks the RmInt1 target site. The isolated plasmid pool obtained by standard alkaline lysis procedure was analyzed by *Xba*I restriction, agarose gel electrophoresis and blotting onto nylon membranes. Further hybridization with IS*Rm2011-2* (spanning positions 562–1025) and RmInt1 (spanning positions 147–594) DIG-labeled probes generated by PCR was carried out and mobility events were identified on the basis of differences in plasmid migration. Hybridizing bands corresponding to homing products (H) and recipient plasmid (R) were analyzed with Quantity One software (Bio-Rad Laboratories) and invasion efficiency of each target site calculated as 100[H/(H+R)]. Homing efficiency was plotted as percentage of wild-type values (reference RmInt1).

### Sequence alignment

Full-length RmInt1 sequences found in different *S. meliloti* strains (Table 1) were aligned, using the MUSCLE algorithm in Geneious R7.1.7.

## Additional Information

**How to cite this article**: Molina-Sánchez, M.D and Toro, N. Inactivation of group II intron RmInt1 in the *Sinorhizobium meliloti* genome. *Sci. Rep.* **5**, 12036; doi: 10.1038/srep12036 (2015).

## Supplementary Material

Supplementary Information

## Figures and Tables

**Table 1:** Full-length RmInt1-like group II introns in different *S.meliloti* strains.

Intron ID^a^	Strain	Host replicon^b^ (accession number)	Identities^c^	Location	Reference
RmInt1	GR4	pRmeGR4b (Y11597)	1884	1–1884	4
S.me_1021_pA_1	1021	pSymA (AE006469)	1877	1024444–1022561	38
S.me_1021_pB_1		pSymB (AL591985)	1879	675027–676910	39
S.me_1021_pB_2			1878	1576500-1574617	
S.me_2011_pA_1	2011	pSymA (CP004138)	1877	1022783-1020900	40
S.me_2011_pB_1		pSymB (CP004139)	1879	675028-676911	
S.me_2011_pB_2			1878	1576514-1574631	
S.me_GR4_Chr_1	GR4	Chromosome (CP003933)	1884	1817775-1819658	5
S.me_GR4_Chr_2			1884	1945831-1947714	
S.me_GR4_Chr_3			1884	3470300-3472183	
S.me_GR4_Chr_4			1884	3590302-3592117	
S.me_GR4_pC_1		pRmeGR4c[pSymA] (CP003936)	1882	551636-553519	
S.me_GR4_pC_2			1884	659540-661423	
S.me_GR4_pC_3			1883	770032-771915	
S.me_GR4_pC_4			1882	893014-894897	
S.me_GR4_pC_5			1884	1070823-1072706	
S.me_SM11_Chr_1	SM11	Chromosome (CP001830)	1880	3047255-3045372	41
S.me_SM11_Chr_2			1880	3677924-3679809	
S.me_SM11_pC_1		pSmeSM11c[pSymA] (CP001831)	1880	25030-26913	
S.me_SM11_pC_2			1880	454897-456780	
S.me_SM11_pC_3			1880	622673-624556	
S.me_SM11_pD_1		pSmeSM11d [pSymB] (CP001832)	1880	954882-956765	
S.me_BL225C_Chr_1	BL225C	Chromosome (CP002740.1)	1883	667127-669010	42
S.me_BL225C_Chr_2			1883	1002942-1004825	
S.me_BL225C_p01_1		pSINME01[pSymA] (CP002741)	1883	659895-661778	
S.me_BL225C_p02_1		pSINME02[pSymB] (CP002742)	1883	32561-34444	
S.me_BL225C_p02_2				189661-191544	
S.me_Rm41_pB_1	Rm41	pSymB (HE995408.1)	1884	1127881-1129140	43
S.me_102F51_pB_1#	102F51	pSymB (DQ898558)	1882	5240-3357	44

^a^Identification code used throughout the text.

^b^The most common name for the megaplasmids is shown in square brackets.

^c^Nucleotides identical to those of the RmInt1 sequence (1884 nt).

^#^Partial sequence of 8766 bp containing an RmInt1-like group II intron.
