# Supplementary material for: Inactivation of group II intron RmInt1 in the Sinorhizobium meliloti genome
Source: Sci Rep. 2015 Jul 9;5:12036. doi: 10.1038/srep12036 (PMC4496777; doi:10.1038/srep12036)
Supplement: Supplementary Information [file srep12036-s1.pdf]

# **Inactivation of group II intron RmInt1 in the *Sinorhizobium meliloti* genome**

María Dolores Molina-Sánchez and Nicolás Toro*

Grupo de Ecología Genética, Estación Experimental del Zaidín, Consejo Superior de Investigaciones Científicas, Calle Profesor Albareda 1, 18008 Granada, Spain

*Correspondence to nicolas.toro@eez.csic.es

**Supplementary figure 1. Alignment of RmInt1 sequences found in different *S. meliloti* strains.**

The RmInt1 sequence (Y11597) was used as a reference and the remaining sequences are detailed in table 1. Colour code corresponded with that described in the caption of figure 1 in the main text. Sequence identities are represented by dots, and bold residues correspond with nucleotide variations. Squared nucleotides identify mutations shared by at least two different *S. meliloti* strains and circled residues correspond with the two point changes studied in this work that severely alter RmInt1 activity. The different domains of RmInt1 are identified above the nucleotide sequence. The translated amino acids sequence of DIV containing the IEP is also annotated, along with the corresponding domains. The catalytic residues of the IEP are highlighted in RT5 domain. Changes in amino acids sequence are marked with an arrow pointing the introduced modification, and silent mutations are underlined.

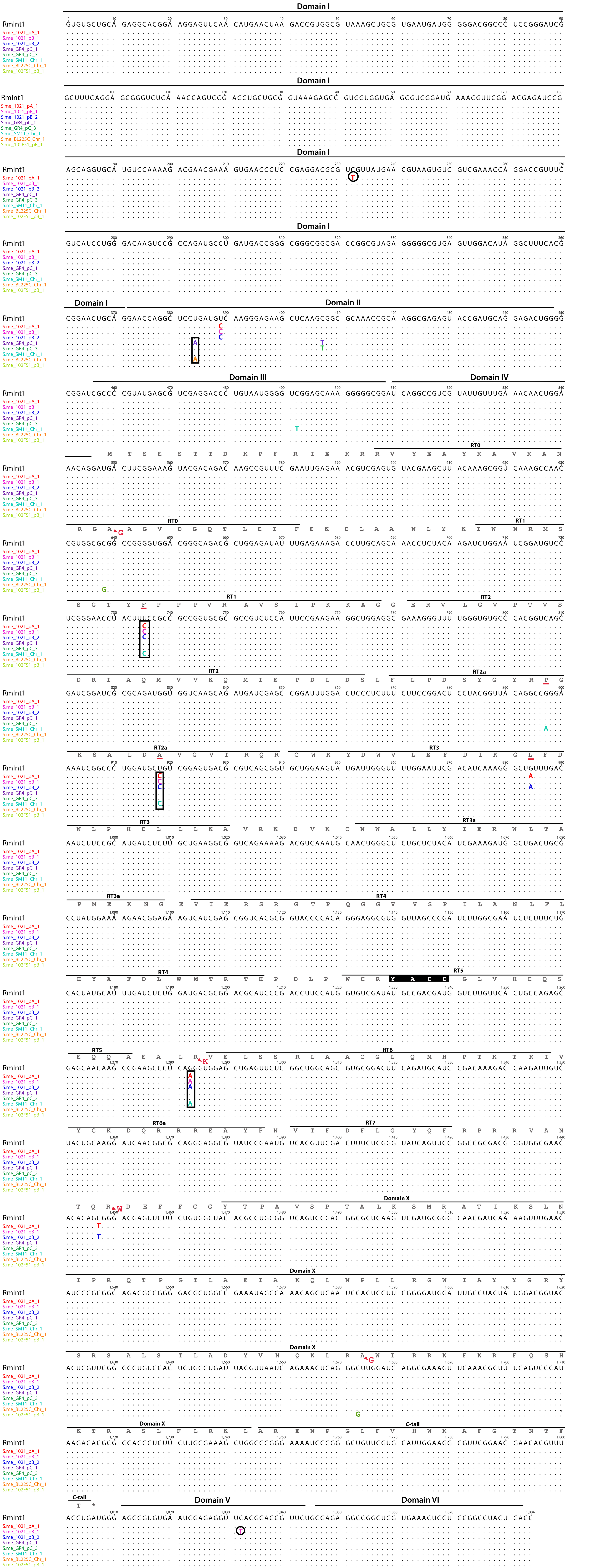

Domain I
Domain II
Domain III
Domain IV
Domain V
Domain VI
Domain X
C-tail
RT0
RT1
RT2
RT2a
RT3
RT3a
RT4
RT5
RT6
RT6a
RT7
RmInt1
S.me_1021_pA_1
S.me_1021_pB_1
S.me_1021_pB_2
S.me_GR4_pC_1
S.me_GR4_pC_3
S.me_SM11_Chr_1
S.me_BL225C_Chr_1
S.me_102F51_pB_1
GUGUGCUGCA GAGGCACGGA AGGAGUUCAA CAUGAACUAA GACCGUGGCG UAAAGCUGCG UGAAUGAUGG GGGACGGCCC UCCGGGAUCG
GCUUUCAGGA GCGGGUCUCA AACCAGUCCG AGCUGCUGCG GUAAAGAGCC GUGGUGGUGA GCGUCGGAUG AAACGUUCGG ACGAGAUCCG
AGCAGGUGCA UGUCCAAAAG ACGAACGAAA GUGAACCCUC CGAGGACGCG UCGUUAUGAA CGUAAGUGUC GUCGAAACCA GGACCGUUUC
GUCAUCCUGG GACAAGUCCG CCAGAUGCCU GAUGACCGGG CGGGCGGCGA CCGGCGUAGA GGGGGCGUGA GUUGGACAUA GGCUUUCACG
CGGAACUGCA GGAACCAGGC UCCUGAUGUC AAGGGAGAAG CUCAAGCGGC GCAAACCGCA AGGCGAGAGU ACCGAUGCAG GAGACUGGGG
CGGAUCGCCC CGUAUGAGCG UCGAGGACCC UGUAAUGGGG UCGGAGCAAA GGGGGCGGAU CAGGCCGUCG UAUUGUUUGA AACAACUGGA
AACAGGAUGA CUUCGGAAAG UACGACAGAC AAGCCGUUUC GAAUUGAGAA ACGUCGAGUG UACGAAGCUU ACAAAGCGGU CAAAGCCAAC
CGUGGCGCGG CCGGGGUGGA CGGGCAGACG CUGGAGAUAU UUGAGAAAGA CCUUGCAGCA AACCUCUACA AGAUCUGGAA UCGGAUGAUC
UCGGGAACCU ACUUUCCGCC GCCGGUGCGC GCCGUCUCCA UUCCGAAGAA GGCUGGAGGC GAAAGGGUUU UGGGUGUGCC CACGGUCAGC
GAUCGGAUCG CGCAGAUGGU GGUCAAGCAG AUGAUCGAGC CGGAUUUGGA CUCCCUCUUU CUUCCGGACU CCUACGGUUA CAGGCCGGGA
AAAUCGGCCC UGGAUGCUGU CGGAGUGACG CGUCAGCGGU GCUGGAAGUA UGAUUGGGUU UUGGAAUUCG ACAUCAAAGG GCUGUUUGAC
AAUCUUCCGC AUGAUCUCUU GCUGAAGGCG GUCAGAAAAG ACGUCAAAUG CAACUGGGCU CUGCUCUACA UCGAAAGAUG GCUGACUGCG
CCUAUGGAAA AGAACGGAGA AGUCAUCGAG CGGUCACGCG GUACCCCACA GGGAGGCGUG GUUAGCCCGA UCUUGGCGAA UCUCUUUCUG
CACUAUGCAU UUGAUCUCUG GAUGACGCGG ACGCAUCCCG ACCUUCCAUG GUGUCGAUAU GCCGACGAUG GUCUUGUUCA CUGCCAGAGC
GAGCAACAAG CCGAAGCCCU CAGGGUGGAG CUGAGUUCUC GGCUGGCAGC GUGCGGACUU CAGAUGCAUC CGACAAAGAC CAAGAUUGUC
UACUGCAAGG AUCAACGGCG CAGGAGGCGG UAUCCGAAUG UCACGUUCGA CUUUCUCGGG UAUCAGUUCC GGCCGCGACG GGUGGCGAAC
ACACAGCGGG ACGAGUUCUU CUGUGGCUAC ACGCCUGCGG UCAGUCCGAC GGCGCUCAAG UCGAUGCGGG CAACGAUCAA AAGUUUGAAC
AUCCCGCGGC AGACGCCGGG GACGCUGGCC GAAAUAGCCA ACAGCUCAAA UCCACUCCUU CGGGGAUGGA UUGCCUACUA UGGACGGUAC
AGUCGUUCGG CCCUGUCCAC UCUGGCUGAU UACGUUAAUC AGAAACUCAG GGCUUGGAUC AGGCGAAAGU UCAAACGCUU UCAGUCCCAU
AAGACACGCG CCAGCCUCUU CUUGCGAAAG CUGGCGCGGG AAAAUCCGGG GCUGUUCGUG CAUUGGAAGG CGUUCGGAAC GAACACGUUU
ACCUGAUGGG AGCGGUGUGA AUCGAGAGGU UCACGCACCG UUCUGCGAGA GGCCGGCUGG UGAAACUCCU CCGGCCUACU CACC
YADD
